# Prediction Models for Pathological Response to Neoadjuvant Chemoradiotherapy in Locally Advanced Rectal Cancer: A Systematic Review and Meta-Analysis

**DOI:** 10.3390/cancers18203359

**Published:** 2026-10-17

**Authors:** Yun Luo, Youping Ding, Wei Liu, Mengying Liu, Qiming Zhao, Minglian Ouyang, You Guo

**Affiliations:** 1 Medical Big Data and Bioinformatics Research Center, First Affiliated Hospital of Gannan Medical University, Ganzhou 341000, China; yydsjzx2018@gmu.edu.cn (Y.L.); dyp19901020@gmu.edu.cn (Y.D.); liuwei@gmu.cn (W.L.); liumengying@gmu.edu.cn (M.L.); zhaoqiming@gmu.edu.cn (Q.Z.); 2 The First School of Clinical Medicine, Gannan Medical University, Ganzhou 341000, China; ouyangminglian@gmu.edu.cn; 3 Ganzhou Key Laboratory of Medical Big Data, Ganzhou 341000, China; 4 Gannan Subcenter for Medical Genomics, National Genomics Data Center, Ganzhou 341000, China; 5 School of Public Health and Health Management, Gannan Medical University, Ganzhou 341000, China

**Keywords:** prediction model, locally advanced rectal cancer, meta-analysis, machine learning, deep learning

## Abstract

**Background**: Accurate pretreatment prediction of pCR is critical for stratifying patients with LARC and selecting candidates for organ-preserving approaches. However, pCR is a postoperative pathological endpoint, whereas watch-and-wait strategies are based on cCR. Despite a rapidly expanding literature encompassing gene expression signatures, MRI radiomics, deep learning, and multi-modal fusion models, no prior systematic review has comprehensively evaluated predictive performance across all modalities using contemporary meta-analytic techniques for diagnostic test accuracy. **Methods**: Systematic review and random- effects meta-analysis of studies developing or validating predictive models for pCR in LARC treated with neoadjuvant chemoradiotherapy (nCRT) were conducted. Summary AUC was estimated using logit-transformed random-effects models. Heterogeneity was quantified by I^2^ and τ^2^. Subgroup analyses were performed according to model modality and validation strategy. Publication bias was assessed with Egger’s linear regression test. Sensitivity analyses were performed using leave-one-out cross-validation. **Results**: Forty-three studies with 67,302 patients were included. The pooled AUC across all models was 0.874 (95% CI: 0.855–0.892), with substantial heterogeneity (I^2^ = 94.3%, τ^2^ = 0.247). Deep learning models showed the highest pooled AUC (0.919, 95% CI: 0.877–0.948), with MRI radiomics (0.885, 95% CI: 0.852–0.911), multi-modal (0.875, 95% CI: 0.813–0.919), gene expression (0.855, 95% CI: 0.797–0.898), and clinical factors (0.820, 95% CI: 0.788–0.849) showing progressively lower pooled estimates. External validation cohorts had a lower pooled AUC (0.864) than internal validation (0.884). Significant publication bias was detected (Egger’s test *p* < 0.001). Leave-one-out sensitivity analysis confirmed robustness of the overall estimate. **Conclusions**: Deep learning and MRI radiomics models demonstrate promising diagnostic accuracy for predicting pCR after nCRT in LARC. However, the predominance of retrospective, single-center designs with limited external validation represents a critical translational gap, and the lack of studies in the total neoadjuvant therapy era limits generalizability. Prospective, multicenter validation studies incorporating standardized endpoints and class imbalance-appropriate metrics are urgently needed.

## Simple Summary

Accurately predicting which patients with locally advanced rectal cancer (LARC) will achieve a pathological complete response (pCR) after preoperative chemoradiotherapy may help risk stratification and trial design. However, pCR is a postoperative pathological endpoint, whereas organ-preserving “watch-and-wait” decisions depend on clinical complete response (cCR). These endpoints are not interchangeable. We conducted a systematic review and meta-analysis of 43 studies covering 67,302 patients. Pooled estimates were numerically highest for deep learning models, followed by magnetic resonance imaging (MRI) radiomics, combined approaches, gene expression signatures, and clinical factors alone. However, between-study heterogeneity was substantial, publication bias was evident, and external validation performance was lower than internal validation performance. These findings should be interpreted cautiously because area under the receiver operating characteristic curve (AUC) alone does not establish clinical utility. Prospective, multicentre validation studies reporting sensitivity, specificity, predictive values, likelihood ratios, calibration, and decision curve analyses are urgently needed.

## 1. Introduction

Colorectal cancer is the third most common malignancy worldwide, with rectal cancer comprising about one-third of cases [1]. For locally advanced rectal cancer (LARC; cT3–T4 and/or node-positive), neoadjuvant chemoradiotherapy (nCRT) followed by total mesorectal excision has been standard for over two decades since CAO/ARO/AIO-94 showed superior local control versus postoperative therapy [2,3]. Pathological complete response (pCR; ypT0N0) is a key surrogate endpoint: pCR occurs in 15–27% after fluorouracil-based nCRT, varying with tumor features, radiotherapy dose/fractionation, chemotherapy, and surgery interval, whereas total neoadjuvant therapy (TNT) can reach 28–38% in selected cohorts, underscoring marked response heterogeneity [4,5].

The seminal observation that a subset of patients achieves a clinical complete response (cCR) following nCRT, characterized by the absence of residual tumor on digital rectal examination, endoscopy, and cross-sectional imaging, has given rise to the organ-preservation strategy known as the watch-and-wait (W&W) approach. Notably, patients with sustained cCR demonstrated non-inferior oncological outcomes compared with surgically treated pCR patients [6]. However, the W&W strategy is predicated on the accurate identification of true complete responders. It is important to emphasize that pCR is a postoperative pathological endpoint, whereas W&W decisions are based on cCR. While these endpoints are correlated, they are not interchangeable. Therefore, prediction of pCR serves as a powerful tool to enrich the pool of potential W&W candidates but cannot replace comprehensive cCR assessment. Discordance between clinical and pathological response assessments remains a significant challenge, with reported rates of undertreatment risk ranging from 4% to 15% depending on the stringency of response criteria employed [7]. This clinical dilemma has intensified the search for robust predictive biomarkers capable of prospectively identifying patients likely to achieve pCR, thereby enabling personalized treatment intensification or de-escalation strategies. A reliable pre-treatment prediction model would serve multiple clinical purposes: identifying patients likely to benefit from nCRT, selecting candidates for organ preservation protocols, and potentially guiding the allocation of more aggressive TNT regimens to non-responders [8].

Over the past decade, a variety of prediction modalities for forecasting pathological response to nCRT in LARC have emerged rapidly, covering molecular, imaging, clinical and machine learning-related domains. However, the comparative diagnostic accuracy of different modalities and the generalizability of these prediction models still remain unclear. A systematic review and meta-analysis of prediction models for pathological response to nCRT in LARC were conducted. This review aims to: (i) quantify the pooled diagnostic accuracy of available prediction models across modalities; (ii) compare performance characteristics between gene expression signatures, magnetic resonance imaging (MRI) radiomics, deep learning, clinical factors, and multi-modal approaches; (iii) evaluate the methodological quality and risk of bias in primary studies using validated quality assessment tools; and (iv) provide evidence-based recommendations for future research priorities and clinical implementation.

## 2. Materials and Methods

This systematic review and meta-analysis were conducted in accordance with the Preferred Reporting Items for a Systematic Review and Meta-Analysis of Diagnostic Test Accuracy Studies (PRISMA-DTA) guidelines [9].

### 2.1. Search Strategy

A comprehensive systematic literature search was performed across four electronic databases: PubMed, Embase, Web of Science, and Cochrane Library. The search strategy was developed in collaboration with an experienced medical information specialist and combined controlled vocabulary terms (MeSH and Emtree) with free-text keywords. Search terms were organized into four conceptual domains: (i) rectal cancer; (ii) neoadjuvant therapy; (iii) response prediction; and (iv) pathological response. Searches were restricted to articles published between January 2005 and December 2025. The full search strings for each database are provided in Supplementary Material S1.

### 2.2. Inclusion Criteria

Participants: Studies involving adult patients (≥18 years) with histologically confirmed LARC (clinical stage II–III, corresponding to cT3–T4 and/or cN1–N2) who received nCRT prior to surgical resection. Studies including a minority of stage I or IV patients were eligible if the predominant population (>70%) met the LARC definition. Studies in which TNT was the exclusive or predominant treatment strategy were recorded separately and excluded from the primary pooled analysis.

Study design: Studies developing, validating, or externally evaluating a multivariable prediction model, including gene expression signatures, MRI radiomics models, deep learning algorithms, clinical nomograms, or multi-modal integrative models, designed to predict pathological response to nCRT prior to treatment initiation or during the treatment course. Single-biomarker studies (e.g., evaluation of a solitary genetic variant or univariable imaging parameter without model construction) were excluded.

### 2.3. Data Extraction

A standardized, piloted data extraction form was developed based on the CHARMS checklist [10]. Two reviewers independently extracted data from each included study, with cross-checking and resolution of discrepancies through consensus discussion. General characteristics were listed for each study and included the authors, year of publication, continent of origin, study design, single-center vs. multi-center status, sample size, and funding sources. Study population characteristics included: Mean or median age, sex distribution, baseline clinical T and N stage distribution, tumor location (distance from anal verge), baseline CEA level, neoadjuvant treatment regimen (radiation dose, chemotherapy type), and interval from nCRT completion to surgery. Prediction model characteristics included: Modality category (gene expression signature, MRI radiomics, deep learning, clinical factors, multi-modal), specific model name or algorithm, number and type of predictors, feature selection method, and model development algorithm.

If published reports contained insufficient detail, the corresponding authors were contacted by email (up to two attempts) to request Supplementary Data.

### 2.4. Quality Assessment

Risk of bias was appraised using a simplified Quality Assessment of Diagnostic Accuracy Studies (QUADAS-2) framework adapted for predictive modeling studies [11]. Four domains were rated as low, unclear, or high risk: (1) patient selection (sampling strategy and sample size adequacy); (2) index test (risk of overfitting based on validation type); (3) reference standard (histopathological pCR); and (4) flow and timing (temporal separation of development and validation cohorts).

We acknowledge that a dedicated tool for prediction model studies, such as PROBAST (Prediction model Risk Of Bias ASsessment Tool), may offer a more comprehensive assessment by specifically addressing issues such as handling of continuous predictors, sample size adequacy, and missing data. The use of an adapted QUADAS-2 represents a limitation of our quality assessment, and future updates of this review should consider adopting PROBAST.

### 2.5. Statistical Analysis

Summary AUC was pooled using a random-effects model with logit transformation to normalize sampling distributions and constrain confidence intervals within [0, 1]. Study-level standard errors were approximated from sample size and AUC using the binomial variance formula. This approach is a widely accepted approximation in diagnostic test accuracy meta-analyses when raw 2 × 2 contingency tables are unavailable. We recognize that this method may underestimate variance compared to bivariate models that jointly model sensitivity and specificity, and therefore our confidence intervals may be somewhat narrow. Between-study heterogeneity was quantified using Cochran’s Q, I^2^, and τ^2^. Prediction intervals were calculated to estimate the range of true effects in future studies. Subgroup analyses were stratified by modality and validation strategy. Publication bias was evaluated with Egger’s linear regression test on logit-transformed AUC against standard error. Sensitivity analysis sequentially excluded each study to assess influence on pooled estimates. All analyses were performed using R software 4.3.3. A two-sided *p* value < 0.005 was considered statistically significant for primary inferences, and *p* < 0.05 was considered exploratory.

## 3. Results

### 3.1. Study Selection

**Figure 1:**
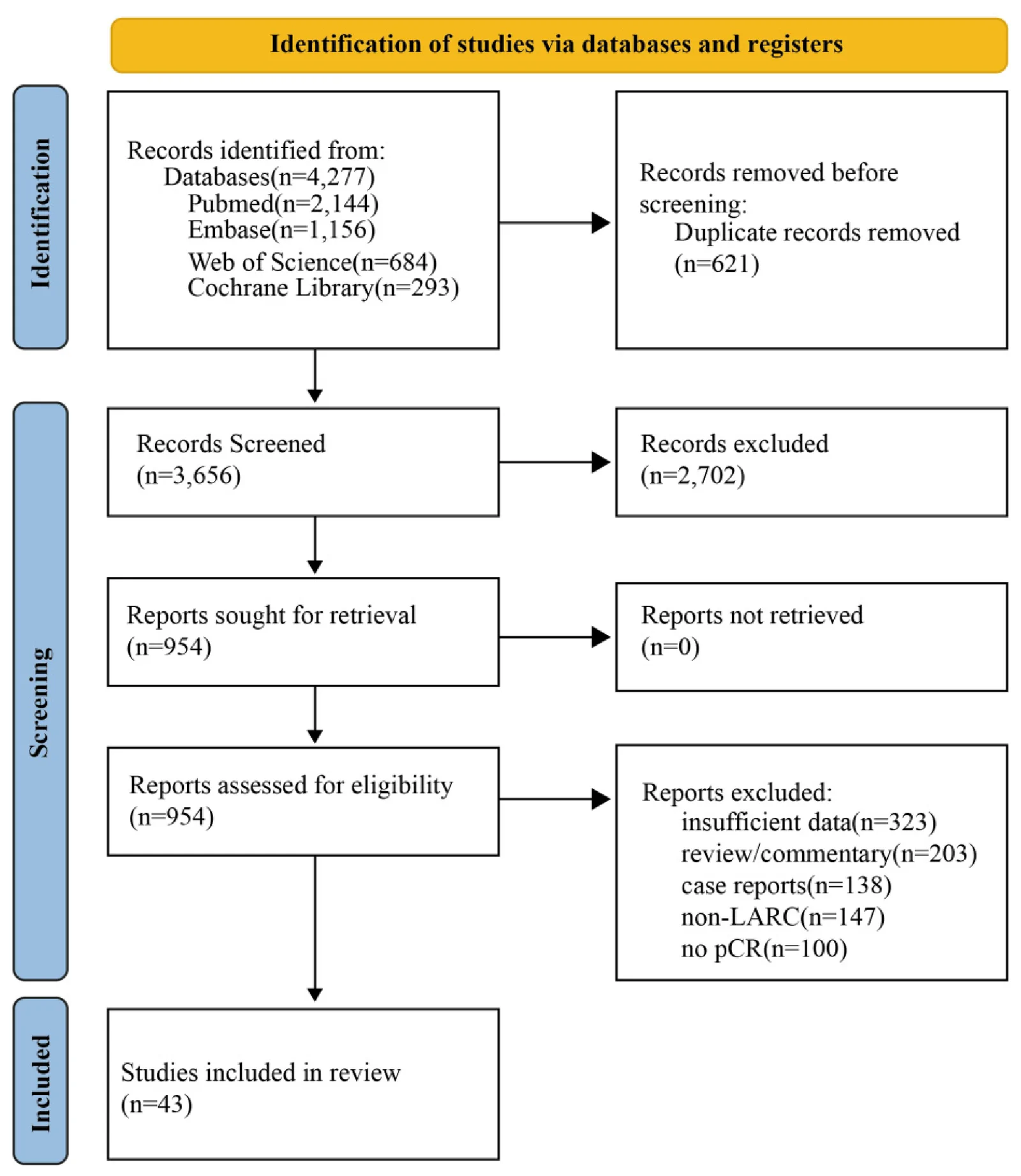
PRISMA flow diagram of study selection. OSF Registries: osf.io/6h2kz.

The systematic search yielded a total of 4277 records across the four electronic databases: PubMed (*n* = 2144), Embase (*n* = 1156), Web of Science (*n* = 684), and Cochrane Library (*n* = 293). Following the removal of duplicate records, 3656 unique records were retained for title and abstract screening. Of these, 954 records met the initial inclusion criteria and were retrieved for full-text assessment. During the full-text review phase, 911 records were excluded. The primary exclusion criteria were as follows: (i) non-original research (e.g., reviews, conference abstracts, letters); (ii) study population not meeting LARC definition or intervention not nCRT; (iii) absence of multivariable prediction model construction or only univariable association analysis; (iv) outcome not pCR; and (v) failure to report AUC or its standard error suitable for meta-analysis. Finally, a total of 43 studies were included in the meta-analysis. The study selection process is shown in the PRISMA flow diagram (Figure 1).

### 3.2. Study Characteristics

A total of 43 studies meeting the inclusion criteria were included, covering 67,302 patients (Table 1) [12,13,14,15,16,17,18,19,20,21,22,23,24,25,26,27,28,29,30,31,32,33,34,35,36,37,38,39,40,41,42,43,44,45,46,47,48,49,50,51,52,53,54]. The distribution of imaging methods is as follows: MRI radiomics (*n* = 13), gene expression (*n* = 9), clinical factors (*n* = 8), deep learning (*n* = 7), and multimodal fusion (*n* = 6). Validation designs included internal validation (*n* = 24), external validation (*n* = 17), and prospective cohort studies (*n* = 2). Notably, six studies had sample sizes smaller than 100 patients, raising concerns about model overfitting.

### 3.3. Risk of Bias Assessment

The reference standard (histopathological pCR) was uniformly rated as low risk across all studies (Table 2). However, patient selection and index-test domains showed considerable methodological limitations. Only 19 studies (44%) employed external or prospective validation, thus demonstrating a low risk of overfitting, while 24 studies (56%) relied on internal or cross-validation, which was associated with a high risk of flow-and-timing bias.

### 3.4. Diagnostic Accuracy

**Figure 2:**
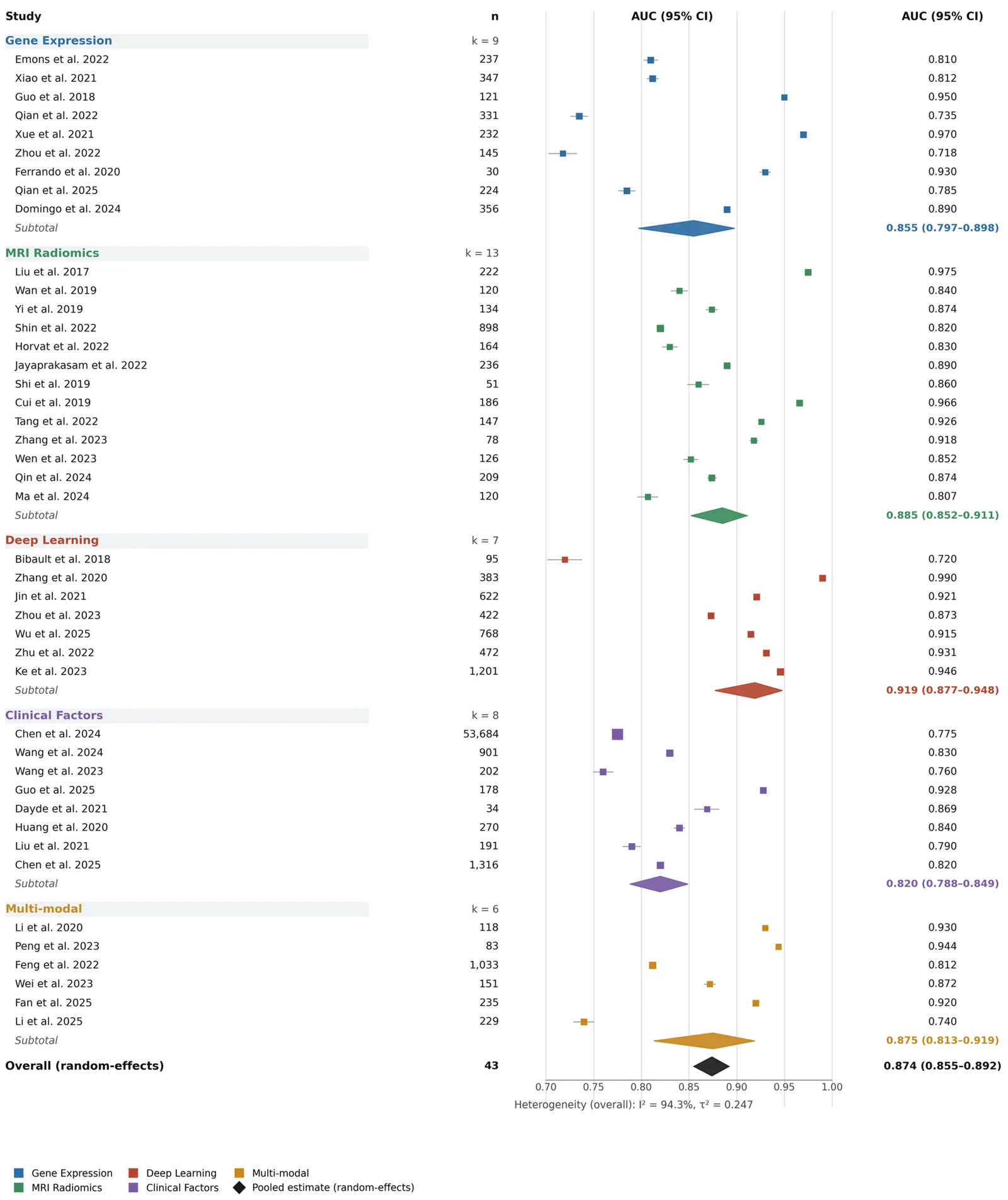
Forest Plot of Diagnostic Accuracy for pCR Prediction in LARC. Note:the studies included in this figure are cited as references [12,13,14,15,16,17,18,19,20,21,22,23,24,25,26,27,28,29,30,31,32,33,34,35,36,37,38,39,40,41,42,43,44,45,46,47,48,49,50,51,52,53,54].

The pooled AUC across all 43 models was 0.874 (95% CI: 0.855–0.892), with substantial between-study heterogeneity (Q = 741.5, *p* < 0.001; I^2^ = 94.3%; τ^2^ = 0.247; prediction interval 0.721–0.949) (Figure 2).

### 3.5. Subgroup Analyses

Subgroup meta-analysis by modality revealed distinct patterns in pooled estimates (Table 3; Figure 2). Deep learning models showed the numerically highest pooled AUC (0.919, 95% CI: 0.877–0.948; *n* = 7), followed by MRI radiomics (0.885, 95% CI: 0.852–0.911; *n* = 13), multi-modal integration (0.875, 95% CI: 0.813–0.919; *n* = 6), gene expression (0.855, 95% CI: 0.797–0.898; *n* = 9), and clinical factors (0.820, 95% CI: 0.788–0.849; *n* = 8). These differences represent observed trends in pooled estimates rather than definitive evidence of comparative superiority, as formal between-subgroup statistical comparisons were not performed due to limited statistical power and uneven group sizes. Heterogeneity remained high across all subgroups (I^2^: 69.3%–92.8%), while MRI radiomics showed relatively lower τ^2^ (0.062) than deep learning (τ^2^ = 0.150). Heterogeneity remained high across all subgroups (I^2^: 79.3%–92.8%), while MRI radiomics showed relatively lower τ^2^ (0.197) than deep learning (τ^2^ = 0.356).

According to validation strategy, internally validated models demonstrated a marginally higher pooled AUC (0.884, 95% CI: 0.859–0.905; I^2^ = 92.9%) than externally validated models (0.864, 95% CI: 0.825–0.896; I^2^ = 93.6%). The two prospective studies yielded a pooled AUC of 0.858 (95% CI: 0.688–0.943; I^2^ = 58.9%).

### 3.6. Publication Bias and Sensitivity Analysis

**Figure 3:**
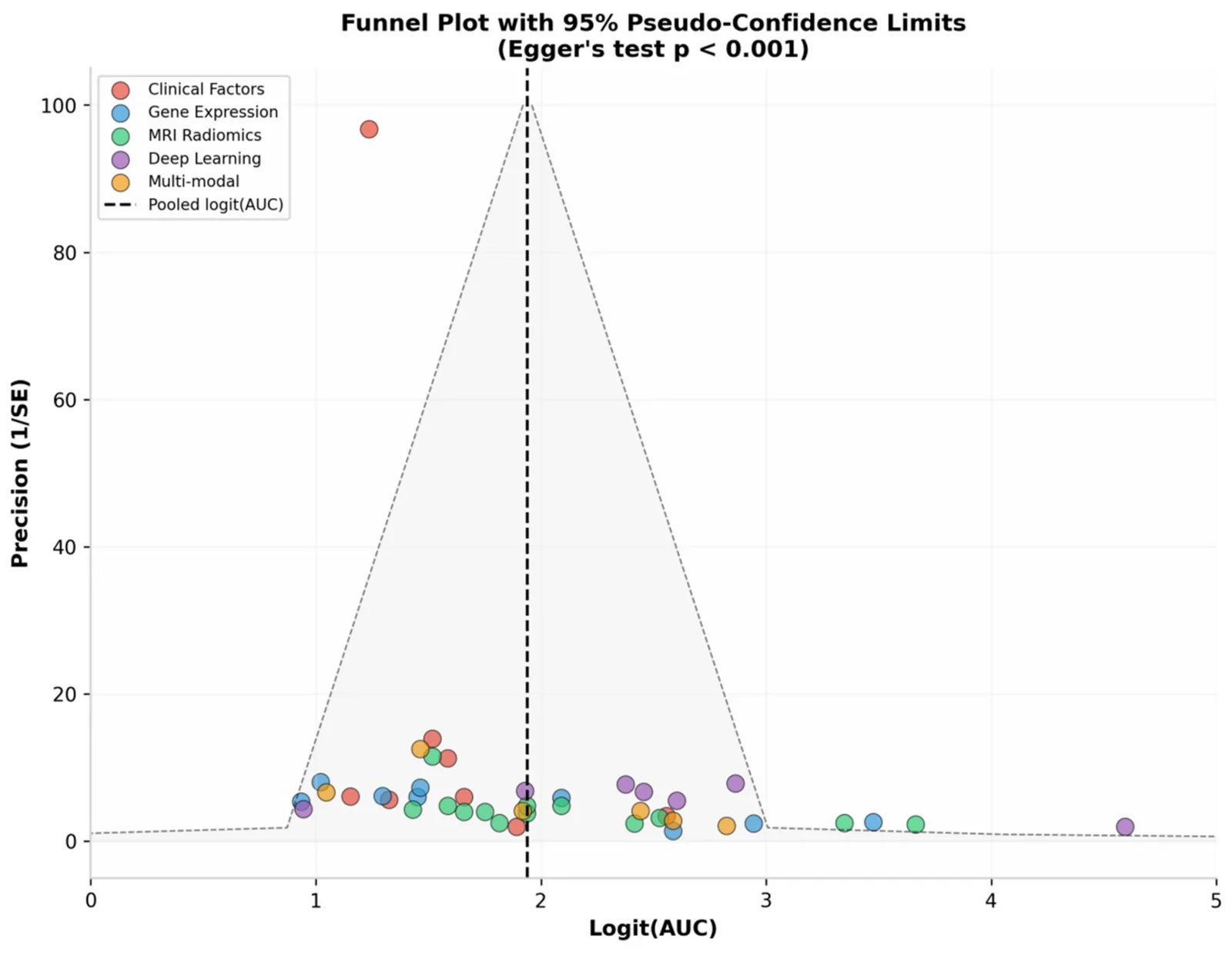
Funnel Plot for Publication Bias Assessment. Logit-transformed AUC plotted against precision (1/SE). The dashed vertical line indicates the pooled logit(AUC). The shaded pseudo-confidence region denotes the 95% expected bounds in the absence of heterogeneity. The observed asymmetry suggests small-study effects favoring high-AUC publications (Egger’s test *p* < 0.001).

Egger’s linear regression test indicated significant asymmetry (intercept = 3.39, SE = 0.51; t = 6.70, *p* < 0.001), suggesting the presence of small-study effects and selective reporting of high-performing models (Figure 3). The funnel plot revealed a relative absence of small, low-accuracy studies.

Leave-one-out sensitivity analysis indicated that the overall combined estimate was stable (range: 0.870–0.877). The most influential individual studies were Zhang et al. 2020 [35] (AUC = 0.990, internal DL), Ke et al. 2023 [40] (AUC = 0.946, external DL), Liu et al. 2017 [21] (AUC = 0.975, internal radiomics), Xue et al. 2021 [16] (AUC = 0.970, external gene expression), and Chen et al. 2024 [41] (AUC = 0.775, internal clinical, *n* = 53,684). Exclusion of the outlier-sized Chen et al. 2024 [41] study reduced I^2^ from 94.3% to 90.7%, with a minimal AUC shift (+0.003).

## 4. Discussion

This systematic review and meta-analysis of 43 studies demonstrates that AI-driven predictive models, particularly deep learning and MRI radiomics, achieve high diagnostic accuracy in identifying pCR after nCRT in LARC. The overall pooled AUC of 0.874 exceeds the performance thresholds typically considered promising for clinical decision support, but the significant heterogeneity, publication bias, and methodological limitations cannot be ignored. The observed differences in pooled AUC across modalities suggest a potential performance gradient. Deep learning models achieved the highest pooled AUC (0.919), likely reflecting their ability to identify hierarchical imaging features beyond human perception [55]. However, this subgroup also showed the greatest heterogeneity (τ^2^ = 0.356), driven by marked discrepancies between internal validation (AUC up to 0.990) and external validation. MRI radiomics offered an attractive balance, with robust accuracy (AUC = 0.885) and comparatively lower between-study variance, suggesting that handcrafted radiomic features may be more reproducible across vendors and protocols. Notably, multi-modal models did not outperform single-modality deep learning or radiomics in pooled estimates, although their prediction intervals were wide and only six studies were available. This finding suggests that current multi-modal fusion strategies may not yet fully exploit the complementary biological and imaging information.

Clinical factor models demonstrated the lowest pooled AUC (0.820) but the smallest τ^2^ (0.060). Although blood-based biomarkers and demographic variables are inherently less predictive, their estimates are more stable across populations. The inclusion of a 53,684 patient national database study (Chen et al. 2024) anchored this subgroup toward real-world generalizability, albeit at lower accuracy [41].

The significant publication bias detected (Egger’s *p* < 0.001) is a major concern. Funnel plot asymmetry indicates that small studies with null or modest results remain unpublished, overstating perceived model performance. Moreover, 56% of included studies relied on internal validation, which systematically overestimates accuracy compared with external validation. The marginal AUC difference between internal (0.884) and external (0.864) cohorts in our analysis likely underestimates true overfitting, given that many external validations originated from the same institution or geographic region.

A critical conceptual issue concerns the relationship between pCR and organ preservation. While the clinical rationale repeatedly links prediction of pCR with selection for W&W strategies, it is essential to recognize that pCR is a postoperative pathological endpoint, whereas W&W decisions are based on cCR. These endpoints are correlated but not interchangeable. Accurate prediction of pCR does not directly establish the ability of a model to safely select patients for non-operative management. Future studies should integrate predictive models into cCR assessment workflows and conduct prospective trials evaluating model-guided W&W strategies against standard care in terms of long-term oncological safety (e.g., local regrowth rate, disease-free survival).

Furthermore, AUC alone provides limited information regarding clinical utility. The absence of sensitivity, specificity, predictive values, calibration, and decision curve measures substantially limits conclusions regarding implementation in clinical practice. The AUC of 0.874 does not guarantee acceptable false-negative rates at clinically relevant thresholds. Future studies must report sensitivity, specificity, PPV, NPV, calibration curves, and decision curve analysis at predefined thresholds.

One of the core challenges of this meta-analysis is the substantial clinical heterogeneity across studies, which extends beyond what could be statistically resolved. Differences in nCRT protocols (radiation dose fractionation, chemotherapy regimens), MRI acquisition parameters (field strength, sequence protocols, reconstruction algorithms), and pCR definitions (ypT0N0 vs. ypT0/TisN0) all contribute to variability in both the true pCR rate and the discriminative ability of prediction models. Additionally, the predominance of Chinese populations (over 60% of studies) may limit generalizability to Western populations with different baseline characteristics and treatment practices. Rather than dismissing these as mere limitations, we emphasize that these unresolved heterogeneities mean our pooled AUC estimates should be interpreted as the average performance across diverse real-world conditions, not as a precise benchmark applicable to all settings. Future research must adopt standardized reporting frameworks and perform pre-planned subgroup analyses or meta-regression on key clinical and technical covariates. Small-sample studies (*n* < 100) are a potential source of the high heterogeneity observed in our analysis. These studies are more prone to producing extreme effect sizes (either inflated or deflated), which can affect the stability of pooled estimates. Our leave-one-out sensitivity analysis suggested that removing individual small-sample studies did not substantially alter the overall estimate, indicating relative robustness. Nevertheless, the risk of overfitting in small-sample studies cannot be dismissed, further emphasizing the need for external validation and large-scale prospective investigations.

Our findings carry direct implications for trial design. To enable safe watch-and-wait selection, predictive models must achieve not only high AUC but also stringent NPV to avoid missing residual disease. Future research should prioritize the following aspects: (1) Prospective, multicenter, and multi-institutional external validation; (2) Standardized reporting of sensitivity, specificity, NPV, PPV, calibration, and decision curve results; (3) Establishing an interpretable framework (such as SHAP values) to enhance the trust of clinicians; (4) Conducting randomized controlled trials to compare the effects of model-based treatment allocation with standard treatment allocation.

Limitations of this meta-analysis include the exclusive reliance on AUC as a summary metric without access to study-level contingency tables, precluding bivariate hierarchical modeling (e.g., HSROC curves). The estimation of standard errors based on sample size and AUC using the binomial variance formula may lead to an underestimation of variance, particularly for studies with extreme AUC values or imbalanced outcomes. Compared with nCRT, TNT regimen differs significantly in treatment intensity, preoperative treatment duration, and pCR rate, which may change the prediction performance of existing predictive models. Future research should focus on the development and validation of predictive models specifically constructed for the TNT era, so as to improve the generalizability and clinical application value of relevant research conclusions. Additionally, significant clinical heterogeneity exists among the studies in terms of nCRT protocols, MRI examination protocols, and pCR definitions, which has not been fully statistically resolved. Finally, the relatively high proportion of the Chinese populations may limit the generalizability of this study to Western populations with different baseline characteristics.

## 5. Conclusions

Artificial intelligence models, especially deep learning and MRI radiomics, demonstrate high diagnostic accuracy for predicting pCR in LARC after nCRT. However, substantial heterogeneity, publication bias, and overfitting risks in internally validated studies highlight the imperative for rigorous, prospective, externally validated, multi-modal prediction systems before integration into clinical workflows. Importantly, pCR prediction is not equivalent to cCR-based selection for W&W. Future studies must report threshold-dependent metrics, calibration, and decision curve analyses to establish clinical utility.

## Figures and Tables

**Table 1:** Characteristics of the 43 included studies predicting pathological complete response to neoadjuvant chemoradiotherapy in locally advanced rectal cancer. All metrics reported from validation cohorts. AUC = area under the curve; REO = relative expression ordering; SINI = systemic inflammation-nutritional index.

Study	Country	*n*	Modality	Predictive Features	ML Algorithm	Validation	AUC
Emons et al. 2022 [12]	USA/ Germany	237	Gene Expression	21-transcript signature	Support Vector Machine	External	0.810
Xiao et al. 2021 [13]	China	347	Gene Expression	15-gene signature	Logistic Regression	External	0.812
Guo et al. 2018 [14]	China	121	Gene Expression	27 gene pairs (REO-based GPS)	Random Forest	External	0.950
Qian et al. 2022 [15]	China	331	Gene Expression	15-gene immune signature	Support Vector Machine	External	0.735
Xue et al. 2021 [16]	China	232	Gene Expression	41 gene pairs (REO-based GPS)	Random Forest	External	0.970
Zhou et al. 2022 [17]	China	145	Gene Expression	5-gene FAM-related signature (LASSO-Cox)	LASSO-Cox regression	External	0.718
Ferrando et al. 2020 [18]	Italy	30	Gene Expression	3-lncRNA signature	LASSO regression	Prospective	0.930
Qian et al. 2025 [19]	China	224	Gene Expression	32 gene pairs (REO-based)	BoostForest	External	0.785
Domingo et al. 2024 [20]	UK	356	Gene Expression	33-gene GBM signature	Gradient Boosting	External	0.890
Liu et al. 2017 [21]	China	222	MRI Radiomics	T2WI + DWI radiomics	LASSO regression	Internal	0.975
Wan et al. 2019 [22]	China	120	MRI Radiomics	CATV + rT2wSI + mrTRG	LASSO regression	External	0.840
Yi et al. 2019 [23]	China	134	MRI Radiomics	T2WI radiomics	LASSO regression	Internal	0.874
Shin et al. 2022 [24]	South Korea	898	MRI Radiomics	Post-nCRT T2WI + ADC radiomics	LASSO regression	Internal	0.820
Horvat et al. 2022 [25]	USA/ Brazil	164	MRI Radiomics	T2WI radiomics	Logistic regression	External	0.830
Jayaprakasam et al. 2022 [26]	USA	236	MRI Radiomics	Mesorectal fat radiomics (T2WI)	Support Vector Machine	Cross- validation	0.890
Shi et al. 2019 [27]	China	51	MRI Radiomics	ROI + radiomics	artificial neural network	Cross- validation	0.860
Cui et al. 2019 [28]	China	186	MRI Radiomics	T2-w + ADC + cT1-w	Logistic regression (nomogram)	Internal	0.966
Tang et al. 2022 [29]	China	147	MRI Radiomics	T2WI radiomics	LASSO regression	Internal	0.926
Zhang et al. 2023 [30]	China	78	MRI Radiomics	T2WI radiomics + LN morphologic	logistic regression (nomogram)	Internal	0.918
Wen et al. 2023 [31]	China	126	MRI Radiomics	pre-nCRT + pre + post-nCRT radscore	logistic regression	Internal	0.852
Qin et al. 2024 [32]	China	209	MRI Radiomics	MC + delta-radiomics	Logistic regression	Internal	0.874
Ma et al. 2024 [33]	China	120	MRI Radiomics	synthetic DKI	Support Vector Machine	Internal	0.807
Bibault et al. 2018 [34]	France	95	Deep Learning	CT radiomics (1683 features)	Deep Neural Network	Internal	0.720
Zhang et al. 2020 [35]	China	383	Deep Learning	DKI deep learning	Convolutional Neural Network	Internal	0.990
Jin et al. 2021 [36]	USA	622	Deep Learning	Longitudinal MRI (multi-task DL, Siamese networks)	Multi-task CNN (Siamese)	External	0.921
Zhou et al. 2023 [37]	China	422	Deep Learning	Multi-sequence MRI with channel attention (CFDL)	CNN + Attention mechanism	External	0.873
Wu et al. 2025 [38]	China	768	Deep Learning	SRADL: 3D ResNet50 + sub-regional radiomics	3D ResNet50 + DL	External	0.915
Zhu et al. 2022 [39]	China	472	Deep Learning	Multi-b-value DWI signature-like pictures	CNN (signature conversion)	Internal	0.931
Ke et al. 2023 [40]	China	1201	Deep Learning	delta-MRI	Multi-task DL(DeepRP-RC)	External	0.946
Chen et al. 2024 [41]	USA	53,684	Clinical Factors	lymphovascular invasion + perineural invasion + CEA + Size of tumor + MSI	Gradient boosting	Internal	0.775
Wang et al. 2024 [42]	China	901	Clinical Factors	SINI index	extreme gradient boosting	External	0.830
Wang et al. 2023 [43]	China	202	Clinical Factors	Hematological indexes	Logistic regression (nomogram)	Internal	0.760
Guo et al. 2025 [44]	China	178	Clinical Factors	CRP + IL-6 + CEA + clinical	Logistic regression (nomogram)	Internal	0.928
Dayde et al. 2021 [45]	USA	34	Clinical Factors	VEGFR3 + EGFR + COX2	Logistic regression	Internal	0.869
Huang et al. 2020 [46]	Taiwan	270	Clinical Factors	Clinical + post-CRT CEA	Artificial Neural Network	Cross- validation	0.840
Liu et al. 2021 [47]	China	191	Clinical Factors	CD163 + MCSF	Cox regression	Internal	0.790
Chen et al. 2025 [48]	China	1316	Clinical Factors	Clinical + DTMS	extreme gradient boosting	Internal	0.820
Li et al. 2020 [49]	China	118	Multi-modal	CT + MRI multi-modal radiomics	Logistic regression	Internal	0.930
Peng et al. 2023 [50]	China	83	Multi-modal	Clinical + radiomics + DL	K-Nearest Neighbor (KNN)	Internal	0.944
Feng et al. 2022 [51]	China	1033	Multi-modal	RAPIDS	Machine learning ensemble	Prospective	0.812
Wei et al. 2023 [52]	China	151	Multi-modal	radiomics + clinical features	Random Forest	External	0.872
Fan et al. 2025 [53]	China	235	Multi-modal	radiomics + clinical features	LASSO + logistic regression	Internal	0.920
Li et al. 2025 [54]	China	229	Multi-modal	Clinical + MRI + dosiomics	logistic regression	External	0.740

**Table 2:** Simplified Quality Assessment (QUADAS-2) of Included Studies.

Domain	Low Risk	Unclear Risk	High Risk
Patient selection	18 (42%)	21 (49%)	4 (9%)
Index test (overfitting)	19 (44%)	21 (49%)	3 (7%)
Reference standard	43 (100%)	0	0
Flow and timing	19 (44%)	0	24 (56%)

**Table 3:** Subgroup Meta-Analysis Results by Modality and Validation Strategy.

Subgroup	Studies (*n*)	Pooled AUC (95% CI)	Prediction Interval	τ^2^	I^2^ (%)	Q statistic	*p* Heterogeneity
Overall	43	0.874 (0.855–0.892)	0.721–0.949	0.247	94.3	741.5	<0.001
Modality							
Clinical Factors	8	0.820 (0.788–0.849)	0.730–0.885	0.060	88.0	58.2	<0.001
Gene Expression	9	0.855 (0.797–0.898)	0.648–0.950	0.309	89.7	78.0	<0.001
MRI Radiomics	13	0.885 (0.852–0.911)	0.754–0.950	0.197	79.3	57.9	<0.001
Deep Learning	7	0.919 (0.877–0.948)	0.763–0.976	0.356	92.8	83.7	<0.001
Multi-modal	6	0.875 (0.813–0.919)	0.690–0.957	0.283	88.6	43.8	<0.001
Validation							
Internal/ Cross-validation	24	0.884 (0.859–0.905)	—	0.236	92.9	—	<0.001
External	17	0.864 (0.825–0.896)	—	0.363	93.6	—	<0.001
Prospective	2	0.858 (0.688–0.943)	—	0.372	58.9	—	0.12

## Data Availability

The original contributions presented in this study are included in the article and Supplementary Material. Further inquiries can be directed to the corresponding author.

## Supplementary Materials

The following supporting information can be downloaded at: https://www.mdpi.com/article/10.3390/cancers18203359/s1.

## Abbreviations

The following abbreviations are used in this manuscript:

3D	Three-dimensional
ADC	Apparent diffusion coefficient
AI	Artificial intelligence
AUC	Area under the receiver operating characteristic curve
CATV	Cylindrical approximated tumor volume
CEA	Carcinoembryonic antigen
CFDL	Channel fusion deep learning
CHARMS	Critical Appraisal and Data Extraction for Systematic Reviews of Prediction Modelling Studies
CI	Confidence interval
CNN	Convolutional neural network
COX2	Cyclooxygenase-2
CRP	C-reactive protein
CT	Computed tomography
cCR	Clinical complete response
DeepRP-RC	Multitask deep learning model for response prediction in rectal cancer
DKI	Diffusion kurtosis imaging
DL	Deep learning
DTMS	Dynamic tumor marker score
DWI	Diffusion-weighted imaging
EGFR	Epidermal growth factor receptor
GPS	Gene pair signature
HSROC	Hierarchical summary receiver operating characteristic
IL-6	Interleukin-6
KNN	K-nearest neighbor
LARC	Locally advanced rectal cancer
LASSO	Least absolute shrinkage and selection operator
LN	Lymph node
lncRNA	Long non-coding RNA
MCSF	Macrophage colony-stimulating factor
MeSH	Medical Subject Headings
MRI	Magnetic resonance imaging
mrTRG	Magnetic resonance tumor regression grade
MSI	Microsatellite instability
nCRT	Neoadjuvant chemoradiotherapy
NPV	Negative predictive value
pCR	Pathological complete response
PPV	Positive predictive value
PRISMA-DTA	Preferred Reporting Items for a Systematic Review and Meta-Analysis of Diagnostic Test Accuracy Studies
PROBAST	Prediction model Risk Of Bias ASsessment Tool
QUADAS-2	Quality Assessment of Diagnostic Accuracy Studies-2
RAPIDS	RAdioPathomics Integrated preDiction System
REO	Relative expression ordering
ResNet50	Residual Network-50
ROI	Region of interest
SE	Standard error
SHAP	SHapley Additive exPlanations
SINI	Systemic Inflammatory-Nutritional Marker Index
SRADL	Deep learning with sub-regional radiomics signatures
SVM	Support vector machine
T2WI	T2-weighted imaging
TNT	Total neoadjuvant therapy
VEGFR3	Vascular endothelial growth factor receptor 3
W&W	Watch-and-wait
