# Supplementary material for: Prediction Models for Pathological Response to Neoadjuvant Chemoradiotherapy in Locally Advanced Rectal Cancer: A Systematic Review and Meta-Analysis
Source: Cancers (Basel). 2026 Oct 17;18(20):3359. doi: 10.3390/cancers18203359 (PMC13702563; doi:10.3390/cancers18203359)
Supplement: Supplementary file 1 [file cancers-18-03359-s001.zip › cancers-4626779-supplementary Material S1.pdf]

Supplementary Material S1: Detailed search strategy

PubMed:

("Rectal Neoplasms"[Mesh] OR "rectal cancer"[tiab] OR "rectal cancers"[tiab] OR "rectal carcinoma*"[tiab] OR "rectal neoplasm*"[tiab] OR "rectal tumor*"[tiab] OR "rectal tumour*"[tiab] OR "rectum cancer*"[tiab]) AND ("Neoadjuvant Therapy"[Mesh] OR "Chemoradiotherapy"[Mesh] OR neoadjuvant[tiab] OR "preoperative chemoradiotherapy"[tiab] OR "preoperative radiotherapy"[tiab] OR "pre-operative chemoradiotherapy"[tiab] OR "preoperative radiation"[tiab] OR "neoadjuvant chemotherapy"[tiab] OR "neoadjuvant chemoradiotherapy"[tiab] OR "neoadjuvant radiotherapy"[tiab] OR "preoperative chemoradiation"[tiab] OR "neoadjuvant chemoradiation"[tiab] OR "total neoadjuvant therapy"[tiab] OR "total neoadjuvant treatment"[tiab] OR TNT[tiab] OR "short-course radiotherapy"[tiab] OR "long-course radiotherapy"[tiab]) AND ("Predictive Value of Tests"[Mesh] OR predict*[tiab] OR model*[tiab] OR nomogram*[tiab] OR "machine learning"[tiab] OR "deep learning"[tiab] OR "artificial intelligence"[tiab] OR radiomic*[tiab] OR "radiomics"[Mesh] OR "neural network*"[tiab] OR "random forest"[tiab] OR "support vector machine"[tiab] OR signature[tiab] OR signatures[tiab] OR classifier*[tiab] OR algorithm*[tiab] OR "risk score"[tiab] OR biomarker*[tiab]) AND ("Pathologic Complete Response"[tiab] OR "complete response"[tiab] OR "complete responder*"[tiab] OR "pathological complete response"[tiab] OR "pathologic response"[tiab] OR "pathological response"[tiab] OR "tumor response"[tiab] OR "tumour response"[tiab] OR "tumor regression"[tiab] OR "tumour regression"[tiab] OR "ypT0"[tiab] OR "ypT0N0"[tiab] OR "tumor regression grade"[tiab] OR "tumour regression grade"[tiab] OR "TRG"[tiab] OR "mrTRG"[tiab]) AND ("2005/01/01"[Date - Publication] : "2025/12/31"[Date - Publication])

Embase:

('rectum cancer'/exp OR 'rectum tumor'/exp OR 'rectal cancer':ti,ab,kw OR 'rectal carcinoma':ti,ab,kw OR 'rectal neoplasm':ti,ab,kw OR 'rectal tumor':ti,ab,kw OR 'rectal tumour':ti,ab,kw OR 'rectum cancer':ti,ab,kw) AND ('neoadjuvant therapy'/exp OR 'cancer chemoradiotherapy'/exp OR 'preoperative radiotherapy'/exp OR 'neoadjuvant':ti,ab,kw OR 'preoperative chemoradiotherapy':ti,ab,kw OR 'preoperative radiotherapy':ti,ab,kw OR 'preoperative radiation':ti,ab,kw OR 'neoadjuvant chemotherapy':ti,ab,kw OR 'neoadjuvant chemoradiotherapy':ti,ab,kw OR 'neoadjuvant chemoradiation':ti,ab,kw OR 'neoadjuvant radiotherapy':ti,ab,kw OR 'preoperative chemoradiation':ti,ab,kw OR 'total neoadjuvant therapy':ti,ab,kw OR 'total neoadjuvant treatment':ti,ab,kw OR 'tnt':ti,ab,kw OR 'short-course radiotherapy':ti,ab,kw OR 'long-course radiotherapy':ti,ab,kw) AND ('prediction'/exp OR 'machine learning'/exp OR 'artificial intelligence'/exp OR 'deep learning'/exp OR predict*:ti,ab,kw OR model*:ti,ab,kw OR nomogram*:ti,ab,kw OR 'machine learning':ti,ab,kw OR 'deep learning':ti,ab,kw OR 'artificial intelligence':ti,ab,kw OR radiomic*:ti,ab,kw OR 'neural network*':ti,ab,kw OR 'random forest':ti,ab,kw OR 'support vector machine':ti,ab,kw OR signature:ti,ab,kw OR signatures:ti,ab,kw OR classifier*:ti,ab,kw OR algorithm*:ti,ab,kw OR 'risk score':ti,ab,kw OR biomarker*:ti,ab,kw) AND ('complete response'/exp OR 'tumor response'/exp OR 'tumor regression'/exp OR 'pathologic complete response':ti,ab,kw OR 'pathological complete response':ti,ab,kw OR 'complete response':ti,ab,kw OR 'complete responder*':ti,ab,kw OR 'pathologic response':ti,ab,kw OR 'pathological response':ti,ab,kw OR 'tumor response':ti,ab,kw OR 'tumour response':ti,ab,kw OR 'tumor regression':ti,ab,kw OR 'tumour regression':ti,ab,kw OR 'ypt0':ti,ab,kw OR 'ypt0n0':ti,ab,kw OR 'tumor regression grade':ti,ab,kw OR 'tumour regression grade':ti,ab,kw OR 'trg':ti,ab,kw OR 'mrtrg':ti,ab,kw) AND [2005-2025]/py

Web of Science:

TS=(("rectal cancer" OR "rectal cancers" OR "rectal carcinoma*" OR "rectal neoplasm*" OR "rectal tumor*" OR "rectal tumour*" OR "rectum cancer*")) AND TS=((neoadjuvant OR "preoperative

chemoradiotherapy" OR "preoperative radiotherapy" OR "pre-operative chemoradiotherapy" OR "preoperative radiation" OR "neoadjuvant chemotherapy" OR "neoadjuvant chemoradiotherapy" OR "neoadjuvant radiotherapy" OR "preoperative chemoradiation" OR "neoadjuvant chemoradiation" OR "total neoadjuvant therapy" OR "total neoadjuvant treatment" OR TNT OR "short-course radiotherapy" OR "long-course radiotherapy")) AND TS=((predict* OR model* OR nomogram* OR "machine learning" OR "deep learning" OR "artificial intelligence" OR radiomic* OR "neural network*" OR "random forest" OR "support vector machine" OR signature OR signatures OR classifier* OR algorithm* OR "risk score" OR biomarker*)) AND TS=("pathologic complete response" OR "pathological complete response" OR "complete response" OR "complete responder*" OR "pathologic response" OR "pathological response" OR "tumor response" OR "tumour response" OR "tumor regression" OR "tumour regression" OR ypT0 OR ypT0N0 OR "tumor regression grade" OR "tumour regression grade" OR TRG OR mrTRG)

Cochrane Library:

#1 MeSH descriptor: [Rectal Neoplasms] explode all trees
#2 (rectal cancer* OR rectal carcinoma* OR rectal neoplasm* OR rectal tumor* OR rectal tumour* OR rectum cancer*):ti,ab,kw
#3 #1 OR #2
#4 MeSH descriptor: [Neoadjuvant Therapy] explode all trees
#5 MeSH descriptor: [Chemoradiotherapy] explode all trees
#6 (neoadjuvant OR preoperative chemoradiotherap* OR preoperative radiotherap* OR preoperative radiation OR neoadjuvant chemotherap* OR neoadjuvant chemoradiotherap* OR neoadjuvant chemoradiation OR neoadjuvant radiotherap* OR preoperative chemoradiation OR total neoadjuvant therap* OR total neoadjuvant treatment OR TNT OR short-course radiotherapy OR long-course radiotherapy):ti,ab,kw
#7 #4 OR #5 OR #6
#8 MeSH descriptor: [Predictive Value of Tests] explode all trees
#9 (predict* OR model* OR nomogram* OR machine learning OR deep learning OR artificial intelligence OR radiomic* OR neural network* OR random forest OR support vector machine OR signature OR signatures OR classifier* OR algorithm* OR risk score OR biomarker*):ti,ab,kw
#10 #8 OR #9
#11 MeSH descriptor: [Pathologic Complete Response] explode all trees
#12 (pathologic complete response OR pathological complete response OR complete response OR complete responder* OR pathologic response OR pathological response OR tumor response OR tumour response OR tumor regression OR tumour regression OR ypT0 OR ypT0N0 OR tumor regression grade OR tumour regression grade OR TRG OR mrTRG):ti,ab,kw
#13 #11 OR #12
#14 #3 AND #7 AND #10 AND #13
#15 Publication Year from 2005 to 2025
#16 #14 AND #15
